# Elucidating connections between the strigolactone biosynthesis pathway, flavonoid production and root system architecture in *Arabidopsis thaliana*

**DOI:** 10.1111/ppl.13681

**Published:** 2022-04-11

**Authors:** Bethany L. Richmond, Chloe L. Coelho, Helen Wilkinson, Joseph McKenna, Pélagie Ratchinski, Maximillian Schwarze, Matthew Frost, Beatriz Lagunas, Miriam L. Gifford

**Affiliations:** ^1^ School of Life Sciences University of Warwick Coventry UK; ^2^ School of Biosciences Birmingham UK; ^3^ Warwick Integrative Synthetic Biology Centre University of Warwick Coventry UK

## Abstract

Strigolactones (SLs) are the most recently discovered phytohormones, and their roles in root architecture and metabolism are not fully understood. Here, we investigated four *MORE AXILLARY GROWTH* (*MAX*) SL mutants in *Arabidopsis thaliana*, *max3‐9*, *max4‐1*, *max1‐1* and *max2‐1*, as well as the SL receptor mutant *d14‐1* and karrikin receptor mutant *kai2‐2*. By characterising *max2‐1* and *max4‐1*, we found that variation in SL biosynthesis modified multiple metabolic pathways in root tissue, including that of xyloglucan, triterpenoids, fatty acids and flavonoids. The transcription of key flavonoid biosynthetic genes, including *TRANSPARENT TESTA4* (*TT4*) and *TRANSPARENT TESTA5* (*TT5*) was downregulated in *max2* roots and seedlings, indicating that the proposed MAX2 regulation of flavonoid biosynthesis has a widespread effect. We found an enrichment of BRI1‐EMS‐SUPPRESSOR 1 (BES1) targets amongst genes specifically altered in the *max2* mutant, reflecting that the regulation of flavonoid biosynthesis likely occurs through the MAX2 degradation of BES1, a key brassinosteroid‐related transcription factor. Finally, flavonoid accumulation decreased in *max2‐1* roots, supporting a role for MAX2 in regulating both SL and flavonoid biosynthesis.

## 1 INTRODUCTION

As sessile organisms, plants require roots to anchor themselves, absorb water and nutrients and interact with microbiota in the rhizosphere. Soils are inherently heterogenous, with varying water and nutrient levels. The architecture of the root system exhibits high developmental plasticity to adapt to these environmental stressors. The primary and lateral roots (LRs) of plants are able to modify their directionality and growth for more effective nutrient mining, known as ‘root foraging’ (Motte & Beeckman, 2019; Waidmann et al., 2020). On a finer scale, root hairs (RHs) extending from root epidermal cells also finetune the uptake of nutrients and water through increasing root surface area (Gahoonia & Nielsen, 2003). RH growth and development is the result of dynamic interplay within a gene regulatory network, which integrates both developmental and environmental signals (Shibata & Sugimoto, 2019).

As with aboveground growth, phytohormones—and the crosstalk between them—are heavily involved in coordinating root system architecture in response to endogenous and exogenous signals. For example, auxin is a positive regulator of both LR and RH growth, and strigolactones (SLs) functionally interact with the transport of auxin by affecting the abundance and polarisation of PIN‐FORMED (PIN) auxin efflux carriers (Bennett et al., 2016; Shinohara et al., 2013; Weijers & Wagner, 2016).

Despite being the most recently discovered class of phytohormones, SLs are already known to have major functions in plant development and plant‐microbial communications (Bhoi et al., 2021). One of the first of many identified roles of SLs in plant development was the finetuning of shoot architecture, hence the name “MORE AXILLARY GROWTH” (MAX) given to some of the key *Arabidopsis thaliana* SL biosynthesis genes. It has been suggested that SLs inhibit shoot branching, acting antagonistically against the cytokinin promotion of bud growth (Gomez‐Roldan et al., 2008; Umehara et al., 2008). More recently, the SL control of auxin transport has also been implicated in shoot branching regulation (Van Rongen et al., 2019). It has previously been suggested that SL addition has an effect on root system architecture in *A. thaliana*, such as by promoting RH elongation, and decreasing LR growth (Jiang et al., 2016; Kapulnik et al., 2011; Ruyter‐Spira et al., 2011). However, much of previous research regarding phenotypic and transcriptomic responses to SL addition has been performed using the SL analogue *rac*‐GR24, a racemic mixture of two GR24 enantiomers GR24^5DS^ and GR24^ent‐5DS^. It was later revealed that whilst GR24^5DS^ preferentially binds and activates the SL receptor DWARF14 (D14), GR24^ent‐5DS^ can activate the karrikin receptor KARRIKIN INSENSITIVE 2 (KAI2) as a karrikin analogue (Scaffidi et al., 2014). Thus, the use of *rac*‐GR24 means that the exact signalling pathway responsible for certain gene regulatory networks and phenotypes remains elusive.

The process of SL biosynthesis in *A. thaliana* involves the successive action of MAX proteins. First, the carotenoid isomerase DWARF27 (D27) converts all‐*trans*‐β‐carotene into 9‐*cis*‐β‐carotene (Abuauf et al., 2018; Booker et al., 2004). MAX3, also termed carotenoid cleavage dioxygenase‐7 (CCD8), then cleaves 9‐*cis*‐β‐carotene into 9‐*cis‐*β‐10′‐carotenal, which is then further cleaved by MAX4 (aka carotenoid cleavage dioxygenase‐8 [CCD8)]), into carlactone (Sorefan et al., 2003). MAX1 is a Class III cytochrome P450 monooxygenase that catalyses the oxidation and hydroxylation of carlactone in the cytosol, producing SLs (Booker et al., 2005). SLs then move to other cells through PLEIOTROPIC DRUG RESISTANT1 (PDR1) transporters to induce downstream SL responses (Borghi et al., 2016). When SL enters a cell, it is first perceived by the downstream SL receptor and the α/β‐fold hydrolase DWARF14 (D14). MAX2 is an F‐box protein involved in targetting proteins for degradation as a subunit of an S phase kinase‐associated protein 1‐cullin‐F‐box (SCF) E3 ubiquitin ligase (Stirnberg et al., 2007). When SL is bound to D14, D14 can either signal for downstream SL responses or be degraded by SCF^MAX2^, thereby limiting the intensity and duration of SL signalling. SCF^MAX2^ also recruits and degrades other proteins than D14, including SUPPRESSOR OF MAX2 1 (SMAX1) and SMAX1‐LIKE (SMXL) family proteins, which regulate downstream SL responses (Soundappan et al., 2015). It was found that the transcription factor BRI1‐EMS‐SUPPRESSOR 1 (BES1) is both able to interact with and be degraded by MAX2 (Wang et al., 2013). In the presence of SL, evidence suggests that D14 activates BES1 degradation via the SCF^MAX2^ complex, thereby regulating downstream signalling. BES1 is most well‐known as a positive regulator in the brassinosteroid (BR) signalling pathway, directly regulating BR‐responsive gene expression, but it has also recently been found to inhibit flavonoid biosynthesis via targeting key enzymes such as TRANSPARENT TESTA4 (TT4) and TRANSPARENT TESTA5 (TT5) (Liang et al., 2020).

In a very similar mode of action to D14, MAX2 is also able to recruit the karrikin receptor KARRIKIN INSENSITIVE 2 (KAI2) in the presence of karrikin or KAI2‐ligands (Waters & Smith, 2013). The large similarities and overlaps between the SL and karrikin signalling pathways have made research about their specific functions challenging. For example, much of previous work regarding phenotypic and transcriptomic response to SL addition has been performed using *rac*‐GR24. This has created confusion regarding which signalling pathway is responsible for control over which gene regulatory networks and phenotypes, or if an interplay between the two mediates the effects. Karrikin and SL pathways are also both involved in the regulation of flavonoid and anthocyanin biosynthesis, being downregulated in both *max2* and *kai2* plants during drought conditions (Li et al., 2017). In addition, when *d14* plants are treated with *rac*‐GR24, they have enhanced flavonol accumulation.

Within this work, we implicate MAX2‐BES1 degradation in the control of flavonoid biosynthesis, pinpointing an intersection with flavonoid biosynthesis. We found that the *max2* mutant has a different flavonoid profile to the three SL knockdown biosynthesis mutants, suggesting that MAX2 acts as a key regulator of flavonoid biosynthesis.

## 2 MATERIALS AND METHODS

### 2.1 Plant material and growth conditions

*A. thaliana max1‐1*, *max2‐1* (Booker et al., 2005), *max3‐9* (Booker et al., 2004), *max4‐1* (Sorefan et al., 2003) and Col‐0 seeds were kindly donated by Dr Amanda Rasmussen (University of Nottingham, UK). *A. thaliana d14‐1* and *kai2‐2* seeds were kindly donated by Prof Caroline Gujahr (Technical University of Munich, Germany) (Waters et al., 2012). Seeds were sterilised and sown onto sterile ½ MS + 1% sucrose media plates, either containing 1 μM SL analogue r*ac*‐GR24 (Chiralix) resuspended in acetone or only acetone as control. Seeds were then stratified at 4°C in the dark for 2 days before being moved to a controlled environment growth cabinet with a 12/12‐h 21°C light/20°C dark or 16/8‐h light/dark cycle.

### 2.2 Root system architecture analysis

Twelve days after the transfer of seeds to the growth cabinet, root systems were imaged via a flatbed scanner and root samples were flash‐frozen in liquid nitrogen. The images were analysed using Fiji software (Schindelin et al., 2012). Primary root and LR measurements were taken for each seedling: primary root length, average LR length, LR number and LR density (the LR number divided by primary root length). For each genotypes, 5–20 seedlings per biological repeat were measured, and a total of three to four independent biological repeats were conducted. For RH analysis, the images of the first section of fully elongated RHs from the root tip were taken using a Leica MZ FLIII fluorescence stereomicroscope; then, RH length and number counts were measured in a 3 mm area using ImageJ (5–20 seedlings per biological repeat, three to four biological repeats). Measurements were considered statistically significantly different if they had a *p* < 0.05 (analysis of variance and post hoc Tukey or pairwise Wilcoxon rank‐sum test with FDR adjustment).

### 2.3 Diphenylboric acid‐2‐aminoethyl ester staining and confocal microscopy

Ten‐day‐old seedlings grown as above were immersed in a solution containing 0.01% Triton X‐100 and 2.52 mg/ml diphenylboric acid‐2‐aminoethyl ester for 7 min, then rinsed with distilled water for a further 7 min (Sanz et al., 2014). Roots from individual plants were mounted in distilled water and visualised on a Zeiss 880 Laser‐Scanning Confocal Microscope (Carl Zeiss Microimaging) (excitation at 458 nm, emission band pass 500–630 nm, Zeiss EC Plan‐Neofluar ×10 objective lens). Micrographs were taken of optical sections of the epidermal layer. Integrated density measurements were taken using Fiji software (Schindelin et al., 2012) from the beginning of the peak fluorescence in the elongation zone, with this location chosen in order to account for differences in the growth of different genotypes.

### 2.4 RNA extraction, sequencing and data processing

Frozen root tissue (stored at −80°C) from 12‐day‐old seedlings was homogenised by placing sterile beads in sampling tubes and using a Tissuelyzer (Qiagen). RNA was extracted from homogenised material using the Monarch RNA MiniPrep Kit (New England Biolabs) with the recommended on‐column DNase treatment. For RNA sequencing, the quantity and quality of RNA were determined with a Bioanalyzer 2100 RNA 6000 Pico Total RNA Kit (Agilent Technologies). Extracted root RNA from three replicates of samples were sequenced with reactions for paired‐end, unstranded, 150 bp read‐length sequences using an Illumina NovoSeq 6000 by Novogene. The depth of sequencing was determined to be at least 20 million reads per sample. Initial quality control of raw reads was performed using FastQC. Raw reads were then subject to trimming and adaptor removal using Trimmomatic v0.33 (Bolger et al., 2014) with the following parameters: LEADING = 20, TRAILING = 20, SLIDINGWINDOW = 4:20, MINLENGTH = 75 and using PHRED33 quality scores; FastQC was used for quality control of the clean reads. Alignment of trimmed reads was performed using STAR (Dobin et al., 2013) using only paired reads. The index files for STAR were generated with the TAIR10 genome and genome annotation with the sjdbOverhang parameter set to 149 (read length −1). Raw counts data for 32,833 genes in the *A. thaliana* genome were obtained from the alignment files using STAR; raw RNA‐seq data for this manuscript was deposited in the NCBI SRA database (PRJNA784414). Differential expression analysis was carried out using DESeq2 (Love et al., 2014). Genes were considered differentially expressed (DE) if they had a log2 fold change >1 and or <−1, and had a Bonferroni‐corrected *p* < 0.05. Lists of DE genes generated from DESeq2 were queried for overrepresented gene ontology (GO) terms using the ‘enrichGO’ function of the R package clusterProfiler 4.0 (Wu et al., 2021). GO terms were considered overrepresented if they had a *p* < 0.05.

### 2.5 Real‐time PCR

RNA was reverse‐transcribed into cDNA via the ProtoScript II First Strand cDNA Synthesis Kit (New England BioLabs) and stored at −20°C. Primer pairs for qPCR were as follows: TT4F 5′‐CCTAGACCAGGTGGAGATAAAGCT‐3′, TT4R 5′‐TTAGAGAGGAACGCTGTGCAAGAC‐3′ (Zhang et al., 2017), TT5F 5′‐CATCGATCCTCTTCGCTCTC‐3′, TT5R 5′‐AGGTGACACACCGTTCTTCC‐3′ (Jiang et al., 2015). qPCR reactions were performed in 96‐well plates with an Agilent Technologies Stratagene Mx3005P (Real‐time PCR) system using SYBR Green JumpStart *Taq* ReadyMix (Sigma‐Aldrich). Reactions were in 10 μl volumes containing 5 nM of each primer, 1 μl of cDNA and 0.5X SYBR Green mix; each qPCR reaction was run in triplicate. Reactions were performed as follows: 95°C preincubation for 5 min, preceded by 40 cycles of 95°C for 10 s, 58°C or 60°C for 10 s and 72°C for 10 s. Post‐amplification, a dissociation cycle was performed from 65°C to 95°C with continuous acquisition of fluorescence to confirm amplification of single amplicons.

## 3 RESULTS

### 3.1 SL analogue *rac*‐GR24 reduces LR length and number in Col‐0 and all *max* mutants, with the exception of *max2‐1*

In previous work, the *max* mutants had been found to have an aboveground branching phenotype (Gomez‐Roldan et al., 2008; Umehara et al., 2008), but given the links between MAX function and hormone pathways related to root development, such as the auxin response pathway, we were interested in analysing belowground phenotypes (Bennett et al., 2016; Shinohara et al., 2013; Weijers & Wagner, 2016). In order to assess this, SL biosynthesis mutants *max3‐9*, *max4‐1* and *max1‐1*, and the SL‐ and karrikin insensitive mutant *max2‐1* root system architecture was analysed in the absence and presence of *rac*‐GR24 (Table S1).

**FIGURE 1:**
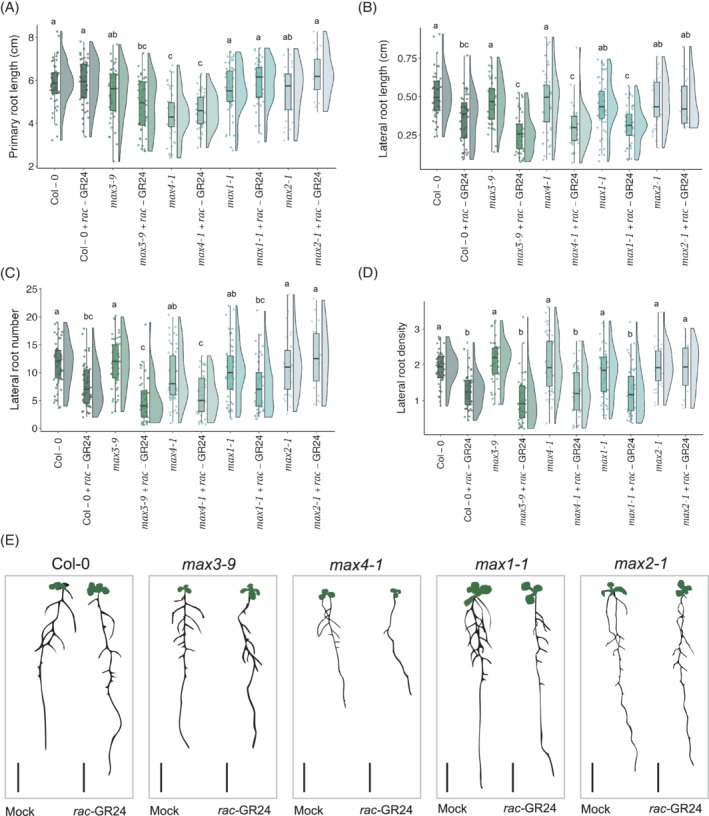
*Rac*‐GR24 reduces the length, number and density of lateral roots in 12‐day‐old Col‐0 and SL biosynthesis mutants, and *max4‐1* plants have a short primary root phenotype that is not rescuable by *rac*‐GR24 treatment. (A) Primary root length (cm) of 12‐day‐old Col‐0, *max3‐1, max4‐1, max1‐1* and *max2‐*1 seedlings after growth on 1 μM *rac*‐GR24 media in 12 h light/12 h dark photoperiod. (B) Lateral root average length (cm) of the same plants, (C) lateral root number and (D) lateral root density. (E) Representative images converted to bitmap for each genotype‐treatment combination, bars represent 1 cm. Different letters indicate statistically significant difference, (ANOVA, post hoc Tukey: *p* < 0.05); (A) *F*_9,429_ = 13.183, (B) *F*_11,535_ = 13.919, (C) *F*_11,535_ = 11.52, (D) *F*_11,535_ = 17.297, 5–20 seedlings per biological repeat, four biological repeats)

Primary root length was not significantly affected by *rac*‐GR24 addition in any of the genotypes. However, *max4‐1* had a significantly shorter primary root length than Col‐0 in both the control and *rac*‐GR24 treatments (*p* values: 9.88E−09 and 6.2E−09, respectively) (Figure 1A–E). Roots treated with *rac*‐GR24 also had significantly lower average LR length for Col‐0 (*p* = 8.19E−05), *max3‐9* (*p* = 3.27E−08), *max4‐1* (*p* = 6.88E−06) and *max1‐1* (*p* = 1.62E−03), but not for *max2‐1* (Figure 1B,E). The number of LRs per plant was significantly lower with GR24 treatment in Col‐0 (*p* = 0.005), *max3‐9* (*p* = 2.06E−09), *max4‐1* (*p* = 2.81E−02) and *max1‐1* (*p* = 0.03) though not in *max2‐1* (Figure 1C,E). LR density also decreased with *rac*‐GR24 addition in Col‐0 (*p* = 5.84E−05), *max3‐9* (*p* = 3.31E−10), *max4‐1* (*p* = 2.81E−07) and *max1‐1* (*p* = 2.56E−03), though not in *max2‐1*. Overall, the *rac*‐GR24 effect is maintained in *max1*, *max3* and *max4*, suggesting it is independent of them, but that there is a MAX2‐dependent role of *rac*‐GR24 in shaping LR development.

### 3.2 SL mutant *max4‐1* has a significantly shorter primary root length compared to Col‐0 under a 12 light/12 dark photoperiod, but not under a 16 light/8 dark photoperiod

Phenotyping of *max* mutants with or without *rac*‐GR24 has been performed previously and some of our results agree with those studies, including the finding that *max4‐1* but not *max2‐1* has a lower density of LRs with GR24 treatment (Kapulnik et al., 2011). However, there are also contradictory results; for example, Villaécija‐Aguilar et al. (2019) found no significant difference in primary root length in *max4‐1* compared to Col‐0 (Villaécija‐Aguilar et al., 2019). When comparing the work, we found that this may be due to differences between the experimental growth conditions, since Villaécija‐Aguilar et al. (2019) use a 16‐h light/8‐h dark photoperiod, and our plants were grown under a 12‐h light/12‐h dark photoperiod. To assess whether day length had an effect on the phenotypes observed, we grew Col‐0 and *max4‐1* again under both 16‐h light/8‐h dark and 12‐h light/12‐h dark conditions, and compared primary root and LR phenotypes as carried out above (Table S1).

**FIGURE 2:**
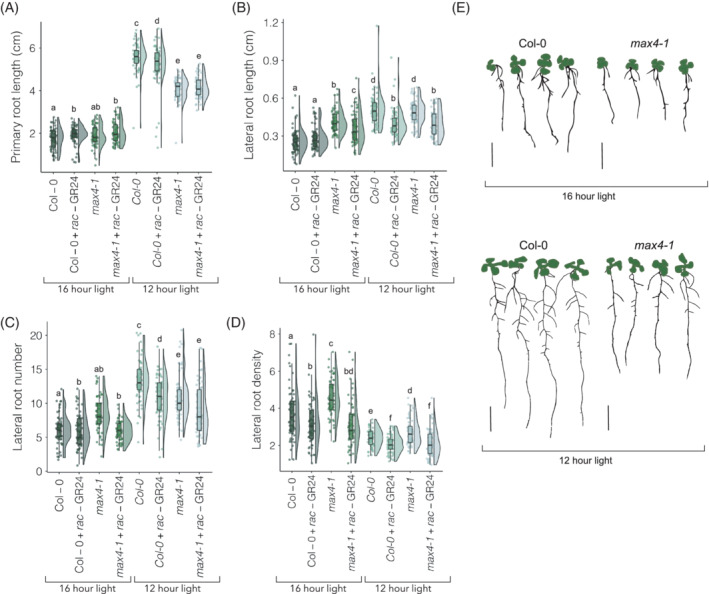
Photoperiod affects the short primary root length phenotype in *max4‐1* plants and this is not rescuable by *rac*‐GR24 treatment. (A) Primary root length (cm) of 12‐day‐old Col‐0 and *max4‐1* seedlings after growth on 1 μM treatment in either a 12 h light/12 h dark photoperiod, or a 16 h light/8 h dark photoperiod, (B) lateral root average length (cm), (C) lateral root number and (D) lateral root density of the same plants. (E) Representative images converted to bitmap; bars represent 1 cm. Different letters indicate statistically significant differences (pairwise Wilcoxon rank sum test [FDR‐adjusted *p* < 0.05]; *n* = 5–20 seedlings per biological repeat, four biological repeats)

In these conditions, all plants grown under a 16‐h light/8‐h dark photoperiod had significantly shorter PRs than those in 12‐h light/12‐h dark. However, we found that primary root length in *max4‐1* was significantly shorter than in Col‐0 in the 12‐h light/12‐h dark photoperiod (*p* = 9.40E−15), a phenotype that was not observed in the 16‐h light/8‐h dark conditions (Figure 2A,E). This suggests that the shorter primary root in *max4‐1* is at least partly daylength‐regulated.

### 3.3 Karrikin receptor mutant *kai2‐2* is not altered in LR length, number or density when treated with *rac*‐GR24

**FIGURE 3:**
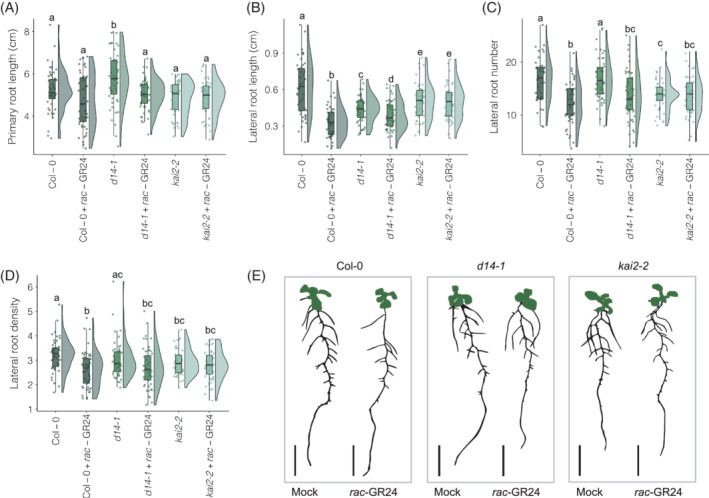
*Rac*‐GR24 reduces the length and number of lateral roots in 12‐day‐old Col‐0 and *d14‐1* roots, but not in *kai2‐2*. (A) Primary root length (cm) of 12‐day‐old Col‐0, *d14‐1* and *kai2‐2* seedlings after growth on 1 μM *rac*‐GR24 media in 12 h light/12 h dark photoperiod, (B) lateral root average length (cm) of the same plants, (C) lateral root number and (D) lateral root density. (E) Representative images converted to bitmap for each genotype‐treatment combination, bars represent 1 cm. Different letters indicate statistically significant difference, determined by pairwise Wilcoxon rank sum test (FDR‐adjusted *p* < 0.05; *n* = 12–20 seedlings per biological repeat, three biological repeats)

We then investigated the *max2‐1 rac*‐GR24 insensitive LR phenotype in more detail in the 12‐h light/12‐h dark photoperiod. As MAX2 interacts with both the SL and karrikin pathways, we analysed the root system architecture of *d14‐1* and *kai2‐2* mutants in the absence and presence of *rac*‐GR24 (Table S1). As with Col‐0, primary root length was not significantly affected by *rac*‐GR24 addition in either *d14‐1* or *kai2‐2* (Figure 3A,E). However, *d14‐1* had a significantly longer primary root length than Col‐0 under mock‐treated conditions (*p* = 0.037). A significant decrease in LR length with *rac*‐GR24 addition was seen in Col‐0 (*p* = 4.1E−09) and *d14‐1* (*p* = 0.024), but not *kai2‐2* (Figure 3B,E). There was also a significant decrease in LR number with *rac*‐GR24 in Col‐0 compared to mock‐treated conditions (*p* = 0.024) and *d14‐1* (*p* = 0.001), but not *kai2‐2*. The lack of LR number and length response to *rac*‐GR24 in *kai2‐2* but not *d14‐1* suggests that this LR developmental regulation in response to *rac*‐GR24 is partly driven by karrikin signalling.

Only Col‐0 had a significant decrease in LR density in response to *rac*‐GR24 compared to mock‐treated conditions (*p* = 0.001). As this effect on LR density is not seen in *d14‐1* or *kai2‐2*, our data agrees with the hypothesis presented by Villaécija‐Aguilar et al. (2019) that KAI2 and D14 coregulate LR density. Under mock conditions, both *d14‐1* and *kai2‐2* had significantly shorter average LR lengths than Col‐0 (*p* = 4.9E−05 and 0.01, respectively), and *kai2‐2* also had significantly lower number and density of LRs per plant (*p* = 0.001 and 0.032, respectively). Thus, both D14 and KAI2 seem to be involved in the LR responses to *rac*‐GR24 and SL signalling.

### 3.4 SL analogue *rac*‐GR24 increases RH number in Col‐0 and *max* mutants, with the exception of *max2‐1*

**FIGURE 4:**
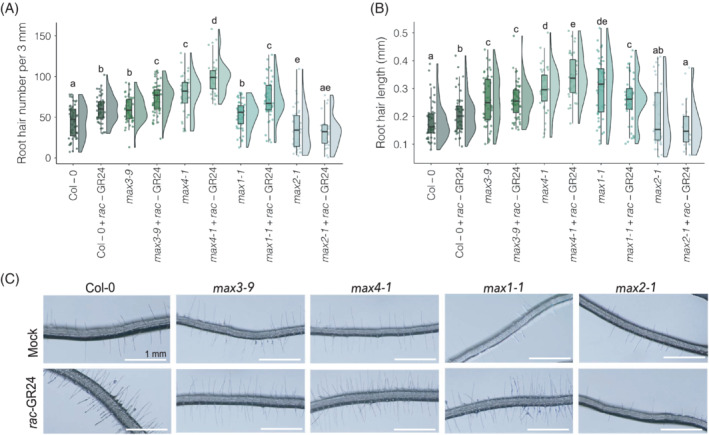
*Rac*‐GR24 increases root hair number, except for in *max2‐1*. (A) Average root hair number (per 3 mm) of Col‐0, *max3‐9*, *max4‐1*, *max1‐1* and *max2‐1* roots after growth on 1 μM *rac*‐GR24 in 12 h light/12 h dark photoperiod and (B) root hair length of the same plants. (C) Representative images, bars represent 1 mm. Different letters indicate statistically significant difference, determined by pairwise Wilcoxon rank‐sum test (FDR‐adjusted *p* < 0.05; *n* = 5–20 seedlings per biological repeat, four biological repeats)

In previous work using a 16‐h light/8‐h dark photoperiod, some authors found no significant RH phenotypes with *rac*‐GR24 addition, even in Col‐0, whereas others found that *rac*‐GR24 addition significantly increases RH length in both Col‐0 and *max4‐1* (Kapulnik et al., 2011; Villaécija‐Aguilar et al., 2019). Thus we next analysed RH number and average length in the *max* mutants (Table S1). We found that RH number increases with *rac*‐GR24 treatment in Col‐0 (*p* = 6E−04), *max3‐9* (*p* = 0.001), *max4‐1* (*p* = 2E−04) and *max1‐1* (*p* = 9.50E−05) but not in *max2‐1* (Figure 4A,C). Also, RHs were significantly more numerous in *max3‐9*, *max4‐1* and *max1‐1* compared to Col‐0 in mock‐treated conditions (*p* = 0.001, 1.10E−08 and 0.042 for *max3‐9*, *max4‐1* and *max1‐1*, respectively) (Figure 4A,C), whilst there are significantly fewer RHs in *max2‐1* (*p* = 0.047).

In line with the phenotypes described by Kapulnik et al. (2011), RH length was found to increase with *rac*‐GR24 addition for Col‐0 (*p* = 0.03) and *max4‐1* (*p* = 0.049) (Figure 4B,C). However, there was a significant decrease in RH length in *rac*‐GR24‐treated *max1‐1* compared to mock‐treated (*p* = 4.07E−02). Also, *max4‐1* and *max1‐1* had longer RHs than Col‐0 without *rac*‐GR24 treatment (*p* = 1.40E−08 and 8.70E−08, respectively) (Figure 4B,C). As *max2‐1* does not exhibit an increase in RH number or length with *rac*‐GR24, these findings support the conclusion that *rac*‐GR24 response in RH number and length is MAX2‐dependent (Kapulnik et al., 2011). It also suggests a role for MAX3, MAX4 and MAX1 in regulating RH number and length.

### 3.5 SL receptor mutant *d14‐1* does not show RH number or length changes with *rac*‐GR24

**FIGURE 5:**
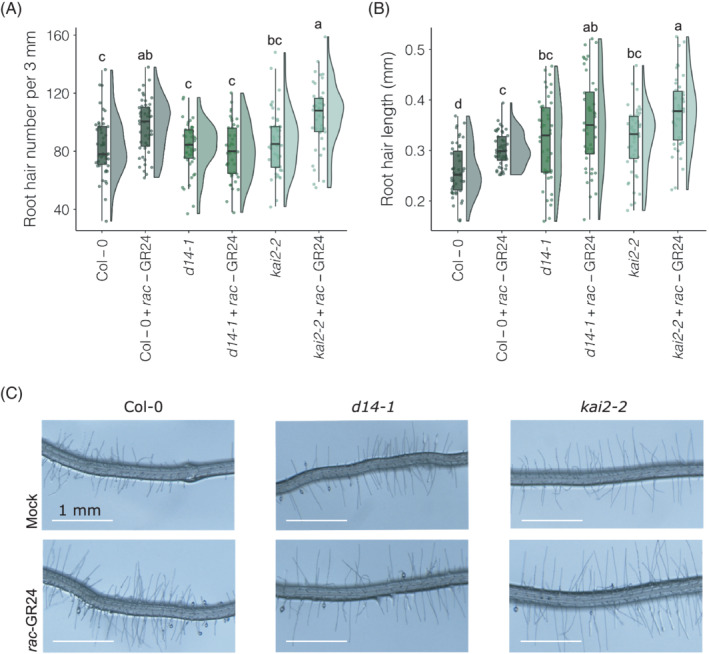
*Rac*‐GR24 treatment increases root hair number and length, except for in *d14‐1*. (A) Average root hair number (per 3 mm) of 12‐day‐old Col‐0, *d14‐1* and *kai2‐2* roots after growth on 1 μM *rac*‐GR24 in 12 h light/12 h dark photoperiod and (B) average root hair length (mm) of the same plants. (C) Representative images, bars represent 1 mm. Different letters indicate statistically significant difference determined by (ANOVA, post hoc Tukey: *p* < 0.05); (A) *F*_5,270_ = 10.203, (B) *F*_5,270_ = 16.211; *n* = 12–20 seedlings per biological repeat, three biological repeats)

As the RH length and number increase in response to *rac*‐GR24 appears to be MAX2‐dependent, we next sought to decipher whether it is also a D14‐ or KAI2‐dependent process. We found that there is an increase in RH number with *rac*‐GR24 addition in Col‐0 (*p* = 0.01) and *kai2‐2* (*p* = 0.001). In addition, there is an increase in average RH length in Col‐0 (*p* = 3.11E−02) and *kai2‐2* (*p* = 2.48E−03). In contrast, *d14‐1* mutants exhibited no change in RH number or length (Figure 5A–C). This implies that the *rac*‐GR24 response in RH length and number is dependent on *D14*, but not *KAI2*. Interestingly, both *d14‐1* and *kai2‐2* plants display longer RHs than Col‐0 under mock conditions (*p* = 3.84E−04 and 1.89E−04, respectively), suggesting that both D14 and KAI2 play a role in RH length regulation.

### 3.6 Triterpenoid biosynthetic genes are commonly downregulated in *max2‐1* and *max4‐1*

**FIGURE 6:**
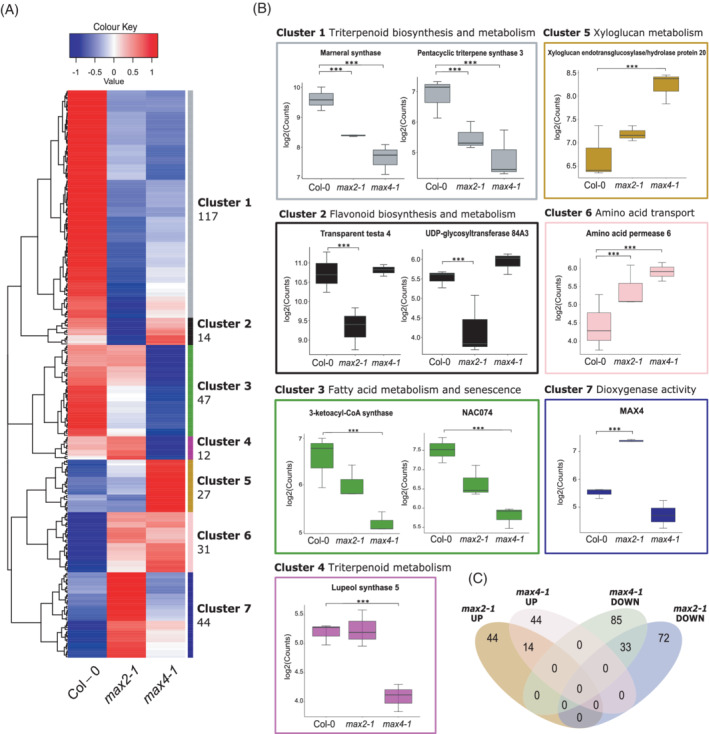
Root transcriptome analysis of 12‐day‐old *max2‐1* and *max4‐1* seedlings implicates metabolic differences in mediating the *max* phenotypes. (A) Heatmap of differentially expressed genes between Col‐0 and either *max2‐1* or *max4‐1* grouped into clusters of similar increased/decreased expression patterns. Genes were considered differentially expressed (DE) if they had a log2 fold change >1 and or <−1, and had a Bonferroni‐corrected *p* < 0.05. (B) Plots of expression values (log_2_(counts)) for selected DE genes per cluster. Asterisks indicate statistically significant difference compared to Col‐0 as indicated by brackets (***p < 0.001; *n* = 20 pooled root samples per biological repeat, three biological repeats). (C) Venn diagram of 292 DE genes in *max2‐1* and *max4‐1* root tissue, showing their shared DE genes and number of upregulated and downregulated DE genes per genotype

To investigate the mechanisms behind the different root phenotypes in *max* mutants, we performed RNA sequencing on root tissue from 12‐day‐old Col‐0, *max2‐1* and *max4‐1* seedlings without *rac*‐GR24 treatment (Table S2). We selected *max4‐1* and *max2‐1* for their different phenotypes; *max4‐1* has a short primary root with longer and more numerous RHs, whilst *max2‐1* has short RHs. We found a total of 292 significantly differentially expressed genes (DEGs) (−1 < FC > 1 and *p* < 0.05) between Col‐0 and either *max2‐1* and *max4‐1* roots (Figure 6A). Using silhouette plotting, DEGs could be grouped into seven clusters, and we then analysed the functions and processes (via GO terms) that were overrepresented within each cluster (Figure 6D) (FDR‐adjusted *p* < 0.05, Table S3).

Given that both *MAX2* and *MAX4* are involved in the SL signalling pathway at different stages, we first investigated common changes between *max2‐1* and *max4‐1* compared to Col‐0 in Cluster 1, which has reduced expression in the *max* mutants, and Cluster 6, which has increased expression in the *max* mutants. Cluster 1, the largest cluster with 117 genes, had an overrepresentation of genes with the associated biological process (BP) GO terms ‘triterpenoid biosynthetic process’ and ‘triterpenoid metabolic process’, as well as related molecular function (MF) GO terms (Table S3). DEGs involved in this cluster include *MARNERAL SYNTHASE* (*MRN1*) and putative *PENTACYCLIC TRITERPENE SYNTHASE 3* (*PEN3*), suggesting that SL biosynthesis and signalling also regulates synthesis of other terpenoid metabolites. Cluster 6 includes 31 genes whose expression is higher in both *max* mutants, with enriched GO terms involved in amino acid transport; these include the *USUALLY MULTIPLE ACIDS MOVE IN AND OUT TRANSPORTERS* (*UMAMIT28*) and *AMINO ACID PERMEASE 6* (*AAP6*).

### 3.7 Fatty acid metabolism is downregulated and xyloglucan metabolism is upregulated specifically in *max4‐1*

Whilst *max2‐1* and *max4‐1* have some similar SL‐related transcriptome profiles (clusters 1 and 6), there are also significant differences between them (DEGs in Clusters 2, 3, 5, 6 and 7). Cluster 3, with 47 genes that have lower expression in *max4‐1* and no change in *max2‐1*, are overrepresented for the GO term involving fatty acid—particularly very long‐chain fatty acid (VLCFA)—and wax metabolism, as well as plant organ senescence (*p* values for terms in Table S3), including two downregulated *3‐KETOACYL‐COA‐SYNTHASE* (*KCS*) genes (*KCS3* and *KCS6*), which catalyse the biosynthesis of VLCFA wax precursors; *KCS6* is found in cluster 1.

Cluster 3 includes DEGs associated with senescence and the cell cycle, including the transcription factor *NAC074*, *BIFUNCTIONAL NUCLEASE 1* (*BFN1*) and *YELLOW‐LEAF‐SPECIFIC GENE 9* (*YLS9*). As these genes have a similar expression pattern to the VLCFA‐related genes, this effect could also be related to a decrease in VLCFA in *max4‐1*, and thus ethylene biosynthesis. The combined role of SLs and ethylene in regulating plant senescence has been studied in Arabidopis leaves (Ueda & Kusaba, 2015) and it is likely to affect root metabolism as well.

In Cluster 4, there are 12 genes with lower expression in *max4‐1* that show no changes in *max2‐1*. This cluster is enriched for GO terms involved in triterpenoid metabolism, including lanosterol synthase, oxidosqualene cyclase and beta‐amyrin synthase activity. One such DEG in this cluster is the terpenoid cyclase gene *LUPEOL SYNTHASE 5* (*LUP5*), which is downregulated in *max4‐1* but not *max2‐1*. This suggests that some SL‐related terpene metabolism is reliant on *MAX4* expression, but not *MAX2*, which is commonly thought to integrate SL downstream responses. It could again be a particular role of carlactone that is required for activating this terpenoid metabolic process. Cluster 5 has 27 genes upregulated in *max4‐1* only, with enriched GO terms relating to the BP GO term ‘xyloglucan metabolism’, and enriched MF terms of polysaccharide and hemicellulose metabolism alongside cell wall biogenesis (*p* values for terms in Table S3), suggesting a link between xyloglucan, SLs and *MAX4* function.

Cluster 7, containing 44 DEGs upregulated in *max2‐1*, had an enriched GO term of ‘dioxygenase activity’ (*p* values for terms in Table S3), including 2‐oxoglutarate (2OG) and Fe(II)‐dependent oxygenase superfamily protein and *GIBBERELLIN 20 OXIDASE 2* (*GA20OX2*). Particularly striking in this cluster was the significant upregulation of *MAX4* in *max2‐1* (*p* = 2.73E−11). This is consistent with the previous finding that SL biosynthesis is upregulated in *max2*, but not *max4‐1* compared to wild‐type (Kohlen et al., 2011).

Overall the gene expression analysis both helped to understand which molecular processes were commonly regulated by *MAX2* and *MAX4* but also distinguished their roles related to root development.

### 3.8 Flavonoid metabolism‐related genes are differentially expressed between *max2‐1* and Col‐0, but not *max4‐1*

Cluster 2 DEGs contains 14 genes that have lower expression in *max2‐1* and are overrepresented for the GO terms ‘flavonoid biosynthesis’, ‘anthocyanin‐containing compound biosynthesis’ and ‘flavonoid metabolism’ (*p* values for terms in Table S3) and none of these 14 genes are differentially expressed in *max4‐1*, signalling that this is a MAX2‐dependent process. Amongst these 14 genes, *TRANSPARENT TESTA 4* (*TT4*) and *UDP‐GLYCOSYLTRANSFERASE 84A3* (*UGT84A3*), two key flavonoid biosynthesis genes, are significantly downregulated in *max2‐1* (*p* = 0.006 and 0.04, respectively). TT4 encodes chalcone synthase, a central enzyme in flavonoid biosynthesis, which catalyses the first committed step by converting its substrate into naringenin chalcone—the precursor to flavonoids and anthocyanins. The mutant *tt4* does not synthesise or accumulate flavonoids (Buer & Muday, 2004). UGT84A3 is a UDP‐glycosyltransferase that glucosylates 4‐coumarate in the phenylpropanoid pathway, which encompasses flavonoid biosynthesis. Unglucosylated 4‐coumarate is required for the primary steps in flavonoid biosynthesis; hence the downregulation of this flavonoid‐limiting step may be a *max2‐1* response to lower levels of flavonoid accumulation. We theorised that the downregulation of flavonoid‐related genes may be a MAX2‐dependent process due to the downregulation of flavonoid metabolism and biosynthesis‐related genes in *max2‐1* but not *max4‐1*. It has been previously shown that MAX2 is capable of degrading the master transcription factor BES1 (Wang et al., 2013). Also, it has been shown that BES1 inhibits CHI and TT4 transcription by repressing the transcription of MYB11, MYB12 and MYB111. These transcription factors activate *CHS* and *CHI* transcription, as well as downstream flavonoid biosynthesis genes *FLAVANONE 3‐HYDROXYLASE* (*F3H*) and *FLAVONOL SYNTHASE1* (*FLS1*) (Liang et al., 2020). We, therefore, hypothesised that in the *max2‐1* mutant, flavonoid biosynthesis might be downregulated due to lack of BES1 degradation.

### 3.9 BES1 targets are over‐enriched amongst *max2‐1* DEGs

MAX2 can degrade BES1, which inhibits flavonoid biosynthesis (Liang et al., 2020; Wang et al., 2013), thus the finding that there is downregulation of flavonoid biosynthesis at the transcript level led to the question of to what extent SL biosynthesis regulates flavonoid biosynthesis, and whether this may involve BES1. Based on previous work showing that BES1 is less readily targeted for degradation in *max2‐1* mutants (Wang et al., 2013), we asked if BES1 target genes were enriched amongst the 163 DEGs between *max2‐1* and Col0. Of the 378 known direct regulatory interactions (based on combining TF binding motifs and regulatory elements data) (Jin et al., 2017; Tian et al., 2020) between BES1 and its interactors in *A. thaliana* root tissue, we identified eight as DEGs in our *max2‐1* dataset, which represented a 3.7‐fold enrichment (hypergeometric test, *p* = 0.001). Of these, three were upregulated: AT3G49620, AT1G56600 (*GALACTINOL SYNTHASE 2*) and AT1G58340, and five downregulated, AT2G01422, AT1G25240, AT1G54970, AT3G55120 (CHI), AT1G06923. A previous work identified putative BES1 target transcription factors through ChIP‐chip and gene expression studies, including AT1G56600 and AT1G06923 (Yu et al., 2011). Using 14‐day‐old gain‐of‐function *bes1‐D* seedlings—in which BES1 accumulates to high levels—Yu et al. (2011) also identified upregulated and downregulated genes compared to wild‐type. Of these, 33 upregulated genes and 38 downregulated genes matched with the 378 direct BES1 targets in roots. Three of the 5 downregulated BES1 direct interactors identified in *max2‐1* seedlings (AT2G01422, AT1G25240, AT1G54970) were also found to be downregulated in *bes1‐D* seedlings (Yu et al., 2011). In comparison, in the *max4‐1* dataset, there was no significant enrichment of root BES1 target genes, further suggesting a direct and specific MAX2‐BES1 link in integrating SL biosynthesis and flavonoid biosynthesis. Furthermore, analysis of microarray data from *bes1‐D* plants found that the most significantly enriched downregulated genes by BES1 were flavonoid biosynthesis‐related (Liang et al., 2020).

**FIGURE 7:**
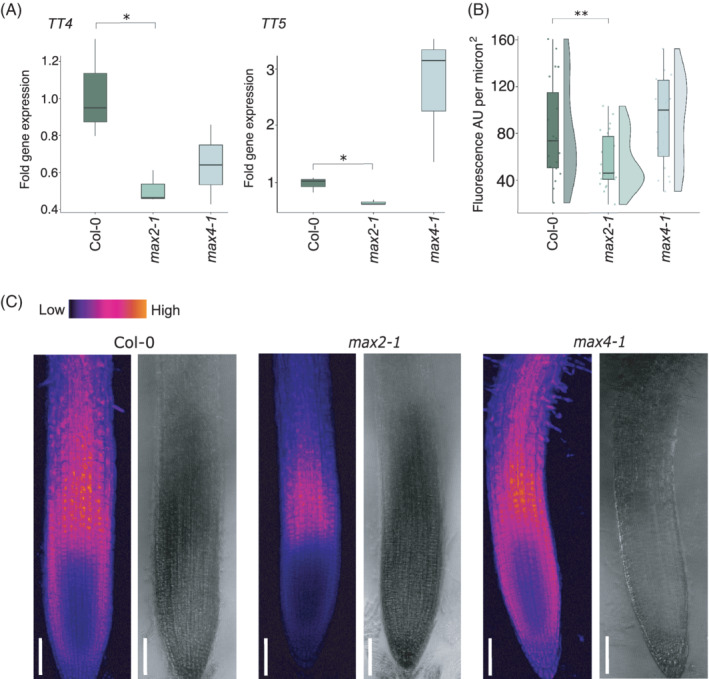
Flavonoid biosynthesis and root accumulation is downregulated in *max2‐1*, but not *max4‐1*. (A) RT‐qPCR was performed using RNA extracted from 12‐day‐old *max2‐1* and *max4‐1* seedlings with primers for *TT5* and *TT5* (*n* = 20 seedlings per genotype per biological repeat, three biological repeats). (B) Ten‐day‐old seedling root tips were stained with 2.52 mg/ml diphenylboric acid‐2‐aminoethyl ester (DPBA), which results in flavonoid fluorescence when excited at 458 nm. Roots were imaged and integrated density measurements were taken from the beginning of the peak fluorescence in the elongation zone as fluorescence arbitrary units (AU) per micron^2^. (C) Representative images of DPBA root tips for Col‐0, *max2‐1* and *max4‐1*; bars represent 100 microns. Asterisks indicate statistically significant difference as indicated by brackets (Student's *t‐*test, *p < 0.05, **p < 0.01)

Using RT‐qPCR on RNA extracted from whole seedlings, we then asked if the BES1‐targeted flavonoid‐related genes identified in our *max2‐1* dataset (*TT4* and *TT5*) were also downregulated in *max2‐1* whole seedlings. Consistent with the downregulation of flavonoid biosynthesis genes in *max2‐1* root tissue, transcripts of both *TT4* and *TT5* were significantly reduced in *max2‐1* whole seedlings, but not *max4‐1* (Figure 7A). This suggests a global effect of lack of *MAX2* on flavonoid biosynthesis gene transcription.

### 3.10 Flavonoid production in *max2‐1* roots is decreased

Given the evidence for downregulated flavonoid‐related transcripts in *max2‐1* in both root tissue and whole plants, we next asked if this leads to decreased flavonoid accumulation in roots of the *max2‐1* mutant. To see if there are earlier changes in *max2‐1* flavonoid accumulation, leading to phenotypic changes, we imaged 10‐day‐old root tips as this is a known area of peak flavonoid accumulation in *A. thaliana* seedlings (Buer et al., 2013). We found that fluorescence in the peak zone in *max2‐1* was significantly reduced (Student's *t*‐test, *p* < 0.002) (Figure 7B,C), confirming that flavonoid accumulation happens less in *max2‐1* root tips compared to wild‐type and *max4‐1*.

## 4 DISCUSSION

SLs are important phytohormones involved in plant development, coordinating developmental cues and stress responses to regulate growth of aerial plant organs (Omoarelojie et al., 2019; Saeed et al., 2017). Here, we add evidence that SLs also play a role in shaping root system architecture. In all *max* mutants, except SL receptor *max2‐1*, we demonstrate that *rac*‐GR24 treatment results in plants with fewer and shorter LRs, concurring with previous studies (Jiang et al., 2016; Kapulnik et al., 2011; Ruyter‐Spira et al., 2011). As *rac*‐GR24 is known to stimulate both SL and karrikin signalling pathways via MAX2, this does not confirm a solely SL‐induced response. Indeed, the formation of junction roots has been attributed to both SL and karrikin signalling through MAX2 (Swarbreck et al., 2020), and so it is likely that LR development is similarly multiregulated. We demonstrate here that, as with *max2‐1*, *rac*‐GR24 addition does not lead to inhibition of *kai2‐2* mutant LR elongation or formation, which is seen for Col‐0, implying that this effect on LR development is a KAI2‐dependent response. However, *rac*‐GR24 did also not affect *d14‐1* nor *kai2‐2* LR density, so it is plausible that both karrikin and SL pathways are responsible for LR *rac*‐GR24 response as discussed by Villaécija‐Aguilar et al. (2019).

As with previous work, we also found that *rac*‐GR24 increases RH length in the *max* mutants *max3‐9* and *max4‐1* and RH number in all SL biosynthesis mutants (Kapulnik et al., 2011). Previous authors have suggested *rac*‐GR24 RH elongation to be karrikin‐driven (Villaécija‐Aguilar et al., 2019). However, we did see a decrease in RH length in *max1‐1* with *rac*‐GR24. This indicates that the accumulation of bioactive SL precursors, such as carlactone, alongside *rac*‐GR24 may influence RH length to some extent. Similarly, if SLs had a positive effect on RH length and density, we would have expected SL biosynthesis mutants to have shorter and less numerous RHs than wild‐type without *rac*‐GR24 treatment. However, we found all *max* biosynthesis mutants to have longer and more numerous RHs than wild‐type. Thus, it is possible that karrikins and SLs both have roles in RH elongation and number. Indeed, whilst *max2‐1* had fewer RHs than wild‐type, we found that neither *kai2‐2* nor *d14‐1* had this phenotype, suggesting a cumulative effect of disrupting both karrikin and SL pathways in *max2‐1*.

Whilst Col‐0 and *kai2‐2* both had an increase in RH number and length with *rac*‐GR24 addition, we confirmed that *d14‐1* did not, suggesting that *rac*‐GR24 response in RHs is dependent on D14 signalling, but not KAI2. Both *kai2‐2* and *d14‐1* had longer average RH lengths than wild‐type, reminiscent of the *max* biosynthesis mutants. Thus, as with LR growth, it is likely that both karrikin and SL signalling contribute to the regulation of RH length.

We also found that *max4‐1* had a short primary root phenotype which was not rescuable by *rac*‐GR24 treatment. Through growing *max4‐1* under two different photoperiods, we found that the *max4‐1* short primary root phenotype is present when plants are grown in a 12‐h light/12‐h dark photoperiod, but not in a 16‐h light/8‐h dark photoperiod. This suggests that the mechanism behind the shorter primary root in *max4‐1* is daylength‐regulated. In support of this hypothesis, it was recently found that D14, which is essential for SL signal transduction and regulation in the SL biosynthesis pathway, is regulated by the circadian clock in rice since overexpression of rice *CIRCADIAN CLOCK ASSOCIATED1* (*OsCCA1*) positively regulated *D14* (Wang et al., 2020a). Given the direct role of *D14* in regulating SL biosynthesis, if *AtD14* is also entrained to the circadian clock, it is likely that this is affecting overall SL or precursor levels, and therefore this could explain the phenotypic differences between the plants.

Through RNA sequencing, we identified a number of processes that are commonly altered in *max2‐1* and *max4‐1*, including amino acid transport. It is possible that this alteration in amino acid transport is a stress response to disturbed SL regulation. Indeed, an increased expression of UMAMIT transporters has been found to induce stress phenotypes, such as stunted growth and increased expression of stress markers in *A. thaliana* (Besnard et al., 2021). The exact mechanism by which increased amino acid transport results in this constitutive stress response is unknown. Given the short primary root phenotype in *max4‐1* but not *max2‐1*, the downregulation of VLCFA‐related genes in *max4‐1* seems of significance. VLCFAs have been implicated in stimulating primary root growth by activating ethylene biosynthesis, which in turn promotes the division of quiescent centre cells (Ortega‐Martínez et al., 2007; Qin et al., 2007). *Kcs* mutants with dysfunctional VLCFA production have significant primary root growth retardation in both *A. thaliana* and barley (Lee et al., 2009; Weidenbach et al., 2015), further supporting that the stunted primary root growth of *max4‐1* but not *max2‐1* could be potentially explained by the lower levels of VLCFA‐related transcripts. As this shorter primary root growth phenotype is present only in *max4‐1*, it could be that this reduction in VLCFA metabolism is a response to low levels of MAX4 and the SL precursor carlactone, as it is a signal molecule able to bind D14 (Abe et al., 2014). Both ethylene and VLCFAs are also implicated in senescence and plant lifespan, as are SLs (Iqbal et al., 2017; Ueda & Kusaba, 2015). Indeed, *MAX4* is upregulated in senescing leaves, and so a similar role for SL in root senescence seems likely (Ueda & Kusaba, 2015).

It has been shown previously that xyloglucan is required for normal RH tip growth and expansion and that interrupting xyloglucan metabolism results in a severe short RH phenotype (Cavalier et al., 2008). In contrast to our finding that *max4‐1* has longer and denser RHs, it is possible that this upregulation in xyloglucan metabolism results in the ‘hairy’ phenotype. Some exemplar xyloglucan‐related DEGs which were significantly upregulated in *max4‐1* but not *max2‐1* include *XYLOGLUCAN ENDOTRANSGLUCOSYLASE/HYDROLASE PROTEIN 15* and *20* (*XTH15* and *XTH20*) as well as *GLUCURONOXYLAN 4‐O‐METHYLTRANSFERASE‐LIKE PROTEIN* (*GXM3*). Given that none of these DEGs are differentially expressed in *max2‐1*, which has no significant difference in its RH average length compared to Col‐0, this data supports the idea that regulation of xyloglucan metabolism could be a SL or carlactone biosynthesis‐related process and is MAX2‐independent. As MAX2 is part of a SL regulatory feedback loop and is able to degrade D14 to limit SL signal transduction, it is plausible that another feedback loop exists whereby overaccumulation of D14 without bound SL results in upregulation of SL biosynthesis genes to maintain basal SL levels. Though, as the other *MAX* SL biosynthetic genes are also expressed in roots, it is interesting that *MAX4* specifically is upregulated in *max2‐1*.

**FIGURE 8:**
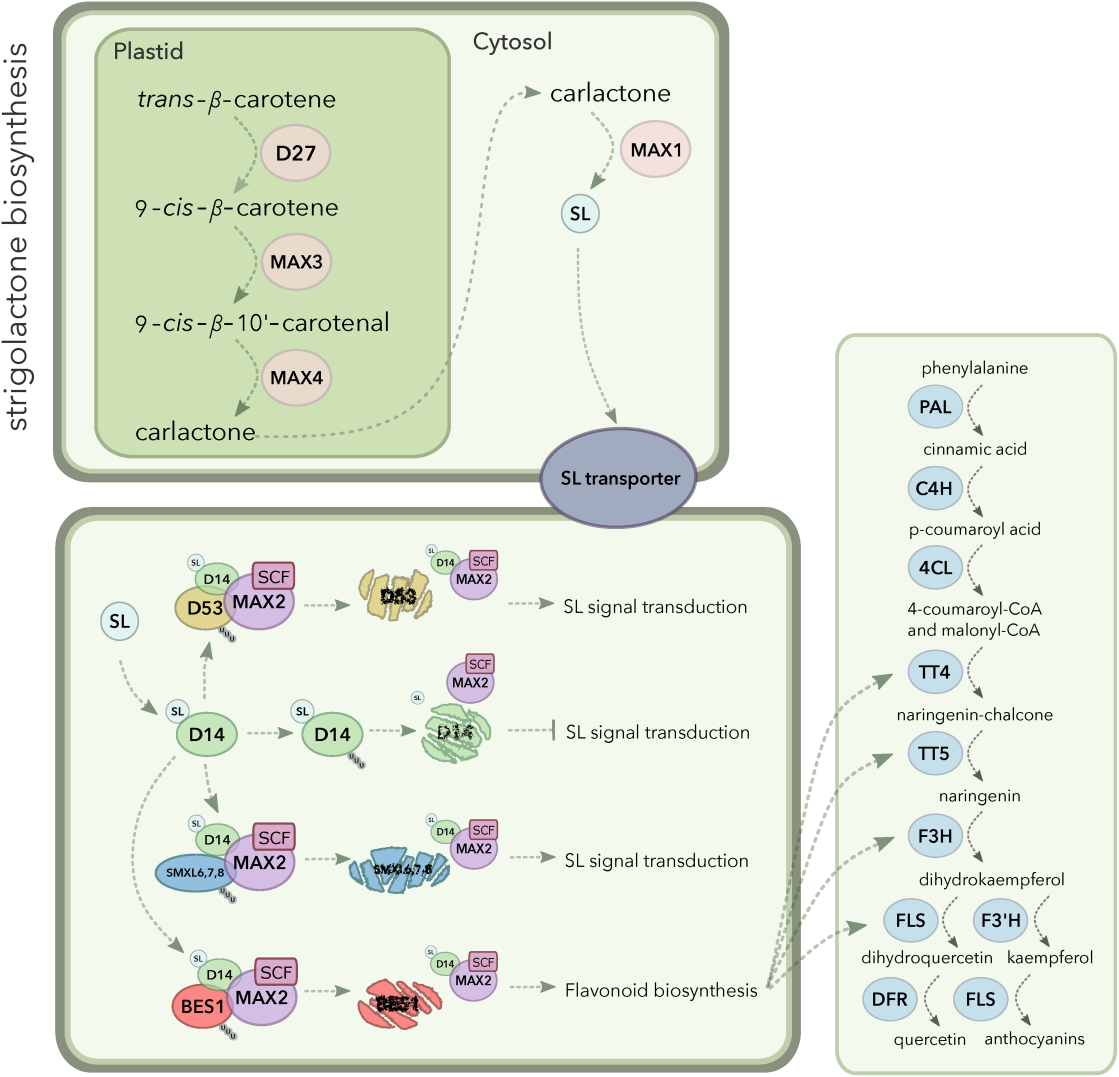
Model showing the hypothetical interaction between strigolactone and flavonoid biosynthesis pathways via MAX2‐BES1, related to the work presented here. BES1 inhibits transcription of *MYB11*, *MYB12* and *MYB111* which, in turn, promotes transcription of key flavonoid biosynthesis genes (*TT4*, *TT5*, *F3H* and *FLS*). BES1 is also able to be degraded by MAX2. With our new phenotypic and transcriptomic data, we hypothesise that the downregulation of flavonoid biosynthesis in *max2‐1* is due to lack of BES1 degradation by MAX2. We propose that plants without a functional MAX2 protein accumulate BES1, leading to over‐inhibition of flavonoid biosynthesis

It is striking that the majority of DE gene clusters were involved in metabolic pathways. SLs, in the roots at least, appear to be part of a metabolite regulatory hub for triterpenoids, flavonoids, fatty acids and xyloglucans. The key to connecting these metabolites seems to be the response to stress since SL production is promoted during nutrient starvation, such as lack of phosphate. Thus the regulation of xyloglucans and fatty acids to shape the root system architecture for optimal root foraging makes sense. The self‐regulation of triterpenoid metabolism could act as a SL feedback loop as well as promote the release of specific triterpenoids into the surrounding soil. As flavonoids are also important components of root‐microbe interactions, co‐regulation of flavonoid biosynthesis under low nutrient stress would be of great benefit to the plant. Equally, flavonoids are important antioxidants that scavenge reactive oxygen species, alleviating general cellular stress. Here, we present a model of co‐regulation of SL and flavonoid biosynthesis via MAX2‐mediated BES1 degradation (Figure 8). The regulation of flavonoid biosynthesis by BES1 has been previously documented; microarray data from *bes1‐D* plants presented the most significantly enriched downregulated genes by BES1 as flavonoid biosynthesis‐related (Liang et al., 2020). Links between SL and flavonoid biosynthesis and MAX2 also have recently suggested, with D14‐specific GR24‐4DO‐treated wild‐type plants showing upregulated flavonoid biosynthesis (Wang et al., 2020b).

Our finding that flavonoids accumulate to a lower level in *max2‐1* supports this, elucidating an exciting link between two key plant metabolic pathways that coordinate root developmental growth and responses to environmental stress.

## AUTHOR CONTRIBUTIONS

*Conceptualisation*: Beatriz Lagunas and Miriam L. Gifford. *Data curation*: Bethany L. Richmond, Chloe L. Coelho, Pélagie Ratchinski, Matthew Frost, Maximillian Schwarze and Helen Wilkinson. *Formal analysis*: Bethany L. Richmond, Pélagie Ratchinski and Chloe L. Coelho. *Investigation*: Bethany L. Richmond. *Methodology*: Beatriz Lagunas, Bethany L. Richmond and Pélagie Ratchinski. *Writing – original draft*: Bethany L. Richmond. *Writing – review and editing*: Bethany L. Richmond, Beatriz Lagunas and Miriam L. Gifford. *Funding acquisition, project administration and supervision*: Miriam L. Gifford. All authors have read and agreed to the published version of the manuscript.

## Supporting information


**Table S1** Phenotypic analysis of the max mutants. (A) Primary and lateral root lengths (cm), lateral root number and density per root and root hair length (mm) and number per 3 mm for Col‐0, *max3‐9*, *max4‐1*, *max1‐1* and *max2‐1* either with or without rac‐GR24 treatment. (B) Primary and lateral root lengths (cm), and lateral root number and density per root of Col‐0 and *max4‐1* in a 12 light/12 dark or 16 light/8 dark photoperiod. (C) Primary and lateral root lengths (cm), lateral root number and density per root and root hair length (mm and number per 3 mm for Col‐0, d14‐1 and kai2‐2 either with or without rac‐GR24 treatment. P values given from T‐test compared to the relevant mock Col‐0 phenotypic measure.

**Table S2** *Arabidopsis thaliana* transcript IDs whose expression was measured via RNAseq in three replicates of Col‐0, *max2‐1* and *max4‐1*. Genes that are differentially expressed (DEGs) between Col‐0, max2‐1 and *max4‐1* are listed together with their gene description, mean count value, log2fold change, log2fold change standard error, Wald's test statistic, P‐value (Wald test of significance) and Benjamin‐Hochberg adjusted P‐value. Genes DE in *max2‐1* compared to Col‐0 or *max4‐1* compared to Col‐0 are noted; genes highlighlighted in yellow are BES1 targets.

**Table S3** GO term enrichment in gene clusters that are DEG in max2/max4. For each cluster number, GO terms of Biological Process (BP) or Molecular Function (MF) that are overrepresented are listed together with the ratios of genes within each cluster (GeneRatio), the Entrez gene IDs, genes with that term in the genome (BgRatio), P value, adjusted P value and q value.

## Data Availability

The raw RNA‐seq data for this manuscript has been deposited in the NCBI SRA database (PRJNA784414, https://www.ncbi.nlm.nih.gov/bioproject/PRJNA784414/).
